# SWEETLEAD: an *In Silico* Database of Approved Drugs, Regulated Chemicals, and Herbal Isolates for Computer-Aided Drug Discovery

**DOI:** 10.1371/journal.pone.0079568

**Published:** 2013-11-01

**Authors:** Paul A. Novick, Oscar F. Ortiz, Jared Poelman, Amir Y. Abdulhay, Vijay S. Pande

**Affiliations:** 1 Department of Chemistry, Stanford University, Stanford, California, United States of America; 2 Department of Computer Science, Stanford University, Stanford, California, United States of America; 3 Department of Biology, Ithaca College, Ithaca, New York, United States of America; 4 Biophysics Program, Stanford University, Stanford, California, United States of America; University of Catania, Italy

## Abstract

In the face of drastically rising drug discovery costs, strategies promising to reduce development timelines and expenditures are being pursued. Computer-aided virtual screening and repurposing approved drugs are two such strategies that have shown recent success. Herein, we report the creation of a highly-curated *in silico* database of chemical structures representing approved drugs, chemical isolates from traditional medicinal herbs, and regulated chemicals, termed the SWEETLEAD database. The motivation for SWEETLEAD stems from the observance of conflicting information in publicly available chemical databases and the lack of a highly curated database of chemical structures for the globally approved drugs. A consensus building scheme surveying information from several publicly accessible databases was employed to identify the correct structure for each chemical. Resulting structures are filtered for the active pharmaceutical ingredient, standardized, and differing formulations of the same drug were combined in the final database. The publically available release of SWEETLEAD (https://simtk.org/home/sweetlead) provides an important tool to enable the successful completion of computer-aided repurposing and drug discovery campaigns.

## Introduction

As the research and development costs needed per approved new chemical entity (NCE) continue to soar[1-3], drug discovery researchers are seeking new strategies aimed at controlling these costs. Repurposing approved drugs to treat new indications is one such strategy, which brings the promise of rapid advancement into clinical trials, pre-determined pharmacokinetic and toxicity profiles, and overall reduced development costs. Many examples of successfully repurposed drugs exist with dramatically lower research price tags in comparison to *de novo* design[4-11]. A second drug discovery strategy aimed at reducing development costs is the utilization of computer-aided drug design, including *in silico* screening[12-14]. These techniques are wide ranging and extremely diverse, but all centered on the goal of filtering a database of molecules to identify promising compounds in hopes of reducing the subsequent costs of experimental validation. Recently, many have seen promising results from combining these two strategies to rapidly identify and advance promising new therapeutic strategies[15-20]. 

 The success of any computational technique is dependent on the quality of data used during the creation of the computational algorithm, and when poor data is used one can only expect poor quality results. A recent report manually evaluated 728 crystal structures used to train well-used docking algorithms, and found only 233 of these crystal structure provided the electron density needed to validate the correctness of the structural model[21]. Of the 233 which did provide density, inspection revealed several errors in the published ligand models - including incorrect element types, incorrect stereochemistry, and even one instance of the wrong ligand being used in the model. In the end, nearly half of the considered structures were deemed to be of insufficient quality for use in developing docking algorithms. It is unreasonable to expect exceptional performance from algorithms trained using such imperfect data.

**Figure 1:**
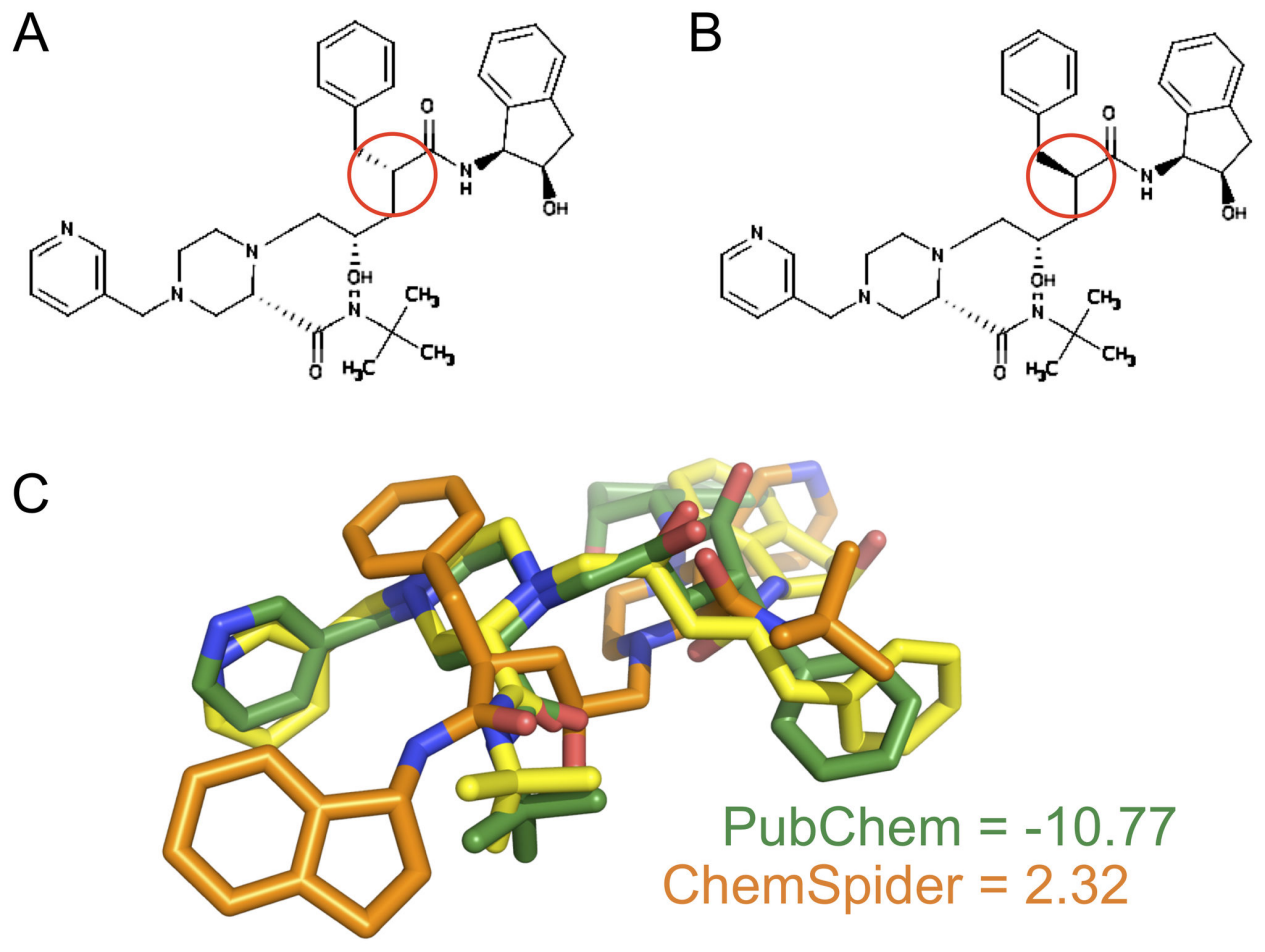
Effect of inaccurate ligand structural information on virtual screening performance. A) and B) Chemical structures for indinavir as depicted by the OpenEye Scientific visualizations program VIDA. The 2D structures returned by ChemSpider (A – ChemSpider ID 4515036) and PubChem (B – PubChem ID 5362440) differ in the stereochemistry of a single chiral center, highlighted by the red circles. C) Effect of the differing structures of indinavir on docking results obtained by the OpenEye Scientific docking program FRED. Ten low energy conformers of each ligand were created with the OpenEye Scientific program Omega, and ligands were docked into the protein structure of HIV protease extracted from the indinavir-bound crystal structure PDB 2R5P. The best scoring pose for the PubChem (green carbons) and ChemSpider (orange carbons) ligands are shown in comparison to the crystallographic ligand (yellow carbons). While the correct structure, obtained from PubChem, scores in the top 6% of all approved drugs, the incorrect structure scores in the bottom 12%.

 It is challenging to find publicly available, complete, and well curated information regarding the molecular structures of the known approved drugs. While several databases are available containing vast amounts of chemical data[22-30], when querying these databases for a given drug conflicting information is occasionally retrieved. As motivation for the present work, consider the HIV protease inhibitor Indinavir. Searching two well used databases, PubChem and Chemspider, for this drug returns PubChem compound 5362440 and ChemSpider compound 4515036[31]. While the structure and IUPAC name suggested on these two databases appear identical, Figure 1a and 1b show the structures perceived by the OpenEye software upon opening the 2D structure files downloaded from each database. Unexpectedly, these two files disagree on the stereochemistry of one of the molecules chiral centers. Figure 1c demonstrates the effect of this difference when Indinavir is docked into its native protein crystal structure (PDB 2R5P) using the OpenEye docking program FRED[32]. While the correct stereoisomer of Indinavir scores in the top 6% of all approved drugs, the incorrect stereoisomer falls to the bottom 12% - demonstrating the sensitivity of such calculations to data integrity. For future efforts utilizing computational screening to repurpose approved drugs, it is essential that a highly accurate database of molecular structures be available.

 We report here our effort to address this issue, and the creation of a database of highly accurate chemical structures of approved drugs. Using an automated tool, we queried several free, public, and well-used chemical databases for reported structures of approved drugs and employed these structures in a consensus building scheme to identify the true structure for every approved drug. In addition to approved drugs, we include the structures of other biologically active and non-toxic molecules (or, molecules for which toxicity profiles are known) such as illegal drugs and chemical isolates from traditional medicinal herbs. We have termed this database the SWEETLEAD database (Structures of Well-curated Extracts, Existing Therapies, and Legally regulated Entities for Accelerated Discovery), and have made it available for public use. 

## Methods

### Resource Evaluation

We first sought to build a tool capable of automating the process of identifying the correct molecular structure for a given drug substance. Prior to building this tool, the free and public resources relevant to such a task were evaluated. It was found the lists of approved drugs, for example from the FDA Electronic Orange Book or the WHO Essential Medicines List, were disseminated as text documents or similar. When structural information was provided, it was frequently in the form of an image of the chemical structure which was not readily convertible to a format amenable for cheminformatics. This constraint required our tool to take as input only the name, or a list of names, of drug substances. We note here, given the ever increasing use of computational tools in drug discovery, the potential utility of regulatory agencies providing structural information as molecular structural files.

 Additionally, we aimed to release SWEETLEAD publicly, and thus required all datasources used to be of a free and public nature. While this eliminates well used but proprietary chemical datasources (such as SciFinder), several publicly available databases have emerged which provide structural and other information about approved drugs. These include PubChem, ChemSpider, DrugBank, KEGG, PharmGKB, ChEBI, and others. Further, several of these databases are either available for download or provide automated querying services, which enable automated querying sufficient for our purposes. In relevance to this work, these databases provide either links to other databases in the form of database IDs (referred to herein as Reference Databases), information describing the chemical structure of a molecule (Structural Databases), or both types of information.

 As indicated above, we have found that when querying various Structural Databases by a drug name, conflicting structural information is sometimes retrieved for a single drug substance. These differences occur most often as conflicting stereochemistry definitions, but included more egregious errors such as incorrect connectivity and incorrect substituents. However, manual inspection revealed that while a single Structural Database may have incorrect structural information, when multiple databases were consulted simultaneously the correct chemical structure was often obvious by simply determining which structure was the most ‘popular.’ That is, the structure present in the highest number of Structural Databases after searching by a drug name was the correct structure. Thus, we decided that a simple ‘rank-by-vote’ scheme, where the structure returned by the highest number of databases was selected as the true structure, would be sufficient to ensure our tool returned the correct structure for a given drug name. Such rank-by-vote approaches have been used to build consensus among conflicting information in other fields, such as virtual screening and gene expression prediction[33-36].

 Finally, we sought to design our algorithm to create a database of structures for use in cheminformatics, and in particular for use in ligand and structure based drug design. It was decided that the most useful end product would be, for a given drug name, a consensus molecular structure, a collection of known synonyms, and relevant database IDs to enable further research when a given drug was identified as a potential hit from virtual screening. Further, since differing formulations such as different salt forms of a drug are equivalent from the standpoint of virtual screening, only the active pharmaceutical ingredient (API) was considered. As a result, multiple specific formulations for a given API have been condensed into a single record in SWEETLEAD.

### Algorithm Workflow

The algorithm developed to create SWEETLEAD is outlined below, but is simplified for ease of explanation. This algorithm was implemented in Python and makes extensive use of the OpenEye Scientific cheminformatics toolkits in addition to freely available Python libraries[37]. The PubChem Power User Gateway (PUG) and ChemSpider WebAPI offer sophisticated automated searching capabilities, while the database information for KEGG, PharmGKB, and DrugBank is available for download and can be manipulated locally. 

**Figure 2:**
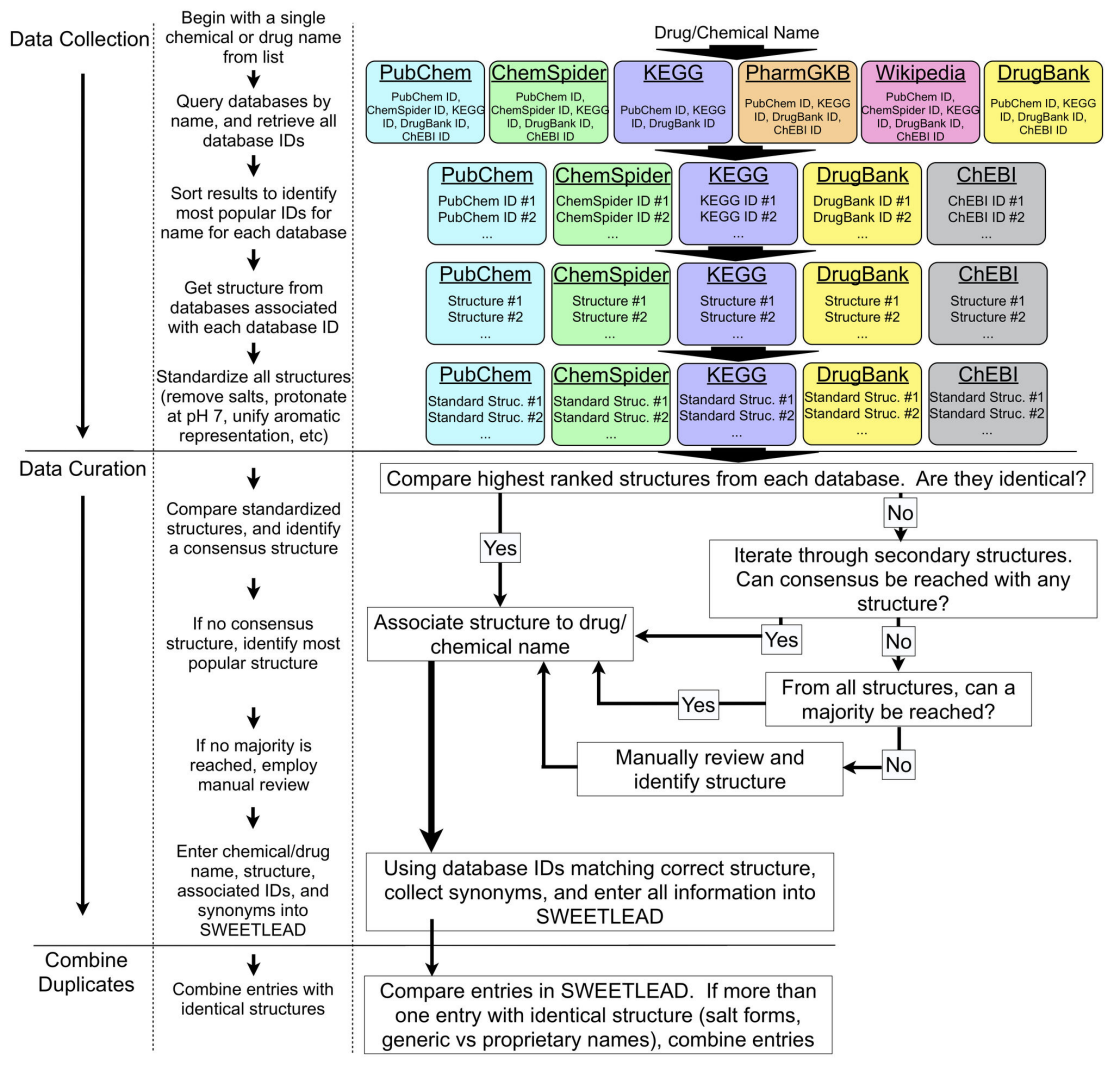
Workflow of the consensus building algorithm. The described process of identifying a correct structure for a given drug begins with a drug or chemical name. In the first stage of the algorithm, the Data Collection stage, several databases are polled by the name and the database IDs linked to that name are retrieved and ranked by frequency each ID was returned (i.e., which ID is ‘most popular’ among databases polled). For each ID returned, the chemical structure associated with that ID is retrieved and standardized (salts removed, standard protonation states and aromaticity models, etc.). In the second stage, the Data Curation stage, the most popular structures from each database are compared. If all structures match, then the structure is assumed to be correct and is assigned to the drug name in the final SWEETLEAD database. If the structures do not match, an iterative cycling through the most popular structures for each database attempts to identify a consensus structure for the drug name. If a consensus or majority structure can not be identified, a manual review is undertaken. Finally, duplicate structures in SWEETLEAD are combined, to allow for numerous brand names and other identifiers for approved drugs.

 The overall workflow of our tool is shown in Figure 2, and can largely be divided into data collection and data curation stages. In the first step of the data collection stage, a drug name is taken as input. Several Reference Databases are then queried using this name, and the internal and external Structural Database IDs from each are collected. The list of Structural Database IDs returned from the previous step is then sorted according to how frequently they were returned; a database ID returned by all queried sources would thus become the ‘most popular’ and highest ranked ID, and less frequently returned IDs would be lower ranked. Next, each Structural Database is then queried by database ID, and the molecular structure associated with each ID is collected (preferably, as a 2-D sdf file). Given that each Structural Database uses a unique processing workflow in creating these structure files, including differing aromaticity models and protonation states, it is then necessary to standardize these structures prior to comparison. Standardization is accomplished by stripping salts and other non-API fragments, assigning specific chirality (when appropriate), applying a common aromaticity model, protonating the API as predicted at pH 7, and finally representing the structure as an isomeric SMILES string. 

 The second stage of the algorithm involves data curation by identifying the correct molecular structure and collecting relevant associated data. First, the highest ranking structures from each Structural Database are compared, as isomeric SMILES strings, to determine whether all suggested structures are identical. If such a consensus is reached, the resulting structure is associated with the input drug name. If the highest ranking structures are not identical, all retrieved structures from the five Structural Databases are compared in an attempt to obtain a consensus. If no consensus is reached, then the number of ‘votes’ each structure receives are tallied and the structure obtaining the most votes is associated with the input drug name. Finally, if no structure receives the majority of votes manual inspection is employed to determine the correct structure by referring to patents filed for the approved medicine. Once the correct structure has been identified, the database IDs linking to that exact structure are collected and recorded. Finally, the synonyms provided by the Structural Databases for each relevant database ID are collected and sorted according to frequency. An entry is then made into our SWEETLEAD containing the drug name, chemical structure, database IDs, and synonyms. Entries which are identical in structure, such as arise from different salt forms of the same API, are combined in the final database. As a concrete example of this process, we have outlined the consensus building scheme for the drug Apomorphine Hydrochloride in the supplementary material (Figure S1). 

## Results and Discussion

Given the input of our tool is a list of drug names, we first sought to obtain lists of approved drugs from several regulatory agencies. We attempted to include approved drug lists from several geographically disperse and highly populated countries with two aims. Firstly, doing so would ensure that the final database represents drugs available for a significant portion of the world population. Secondly, by including geographically diverse countries we hoped that any regional differences in medical training and disease burden would not limit the widespread utility of SWEETLEAD. As such, we have included drugs approved in the USA, India, China, Australia, Brazil, the EMA, the WHO Essential Medicines List, and those listed in the NGCG Pharmaceutical Collection[38]. 

 The original intent of our work was to provide a database of all approved drugs to help facilitate the repurposing efforts occurring in our lab and elsewhere. The benefits of repurposing as a drug discovery strategy are well known, and include the facts that approved drugs a) are known to be physiologically active, b) are relatively well tolerated (or at least have known toxicity), and c) are bioavailable (or at least have known effective routes of administration), among others. We then considered what other chemical substances had these same benefits and may provide useful lead compounds for repurposing efforts. For example, if publicly known, compounds that pass Phase II clinical trials but then are abandoned due to lack of efficacy for their primary indication could still possess the same benefits as approved drugs for use in repurposing campaigns. Further, compounds such as recreational drugs have obvious physiological activity and well known toxicity profiles. Traditional and herbal medicines have been used for centuries for purported medical benefits, and chemical isolates from these substances could also be interesting lead compounds. As such, lists containing these types of chemicals were also obtained and added to the database, and include US scheduled drugs and chemical isolates from traditional Chinese and Ayurvedic medicinal plants.

**Figure 3:**
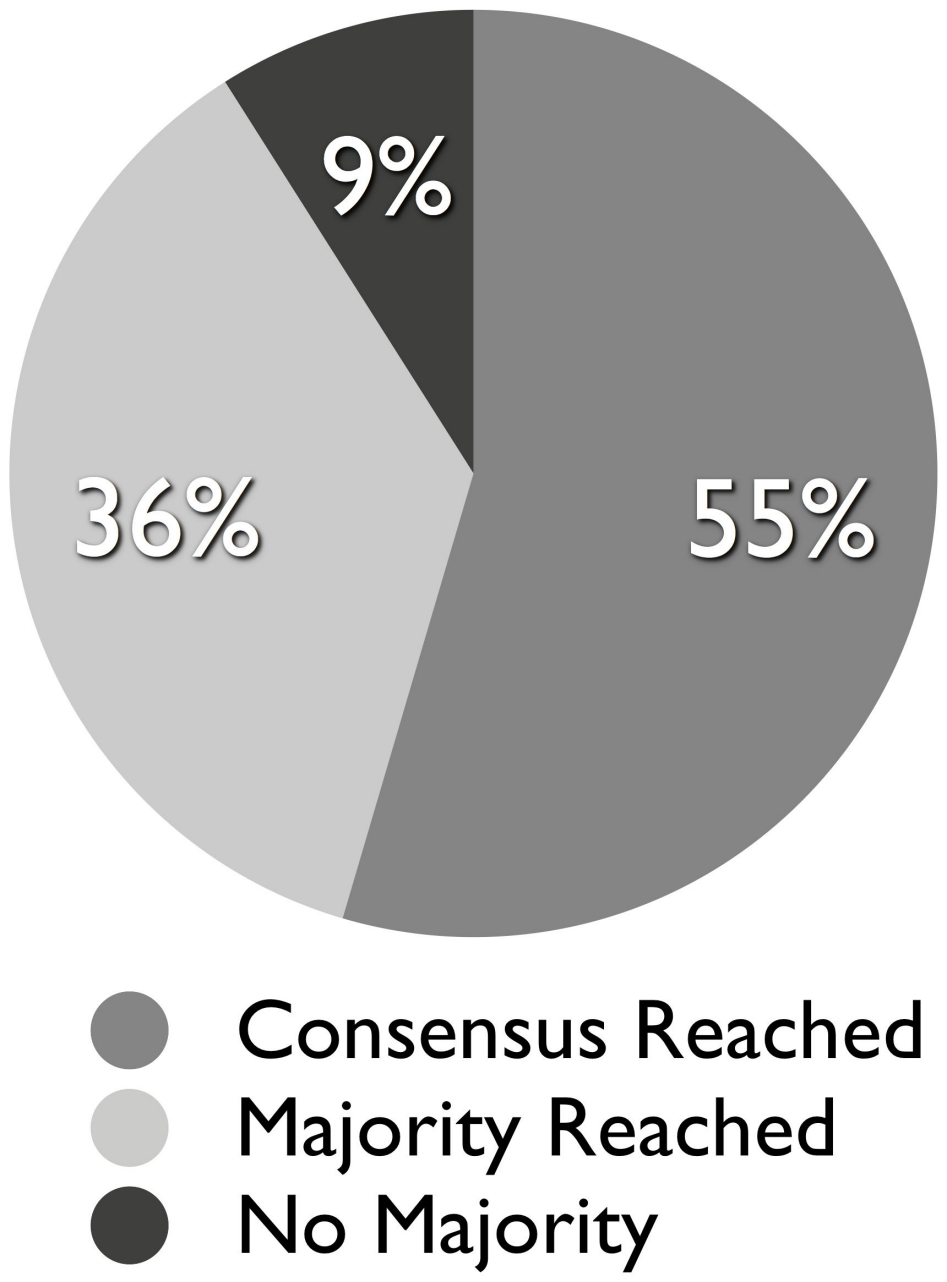
Example outcomes of chemical names input into the SWEETLEAD workflow. For the list of 1996 API names from the FDA orange book, the percentage of compounds is shown for which either a consensus structure, a majority vote structure, or no clear majority structure was identified via the SWEETLEAD algorithm. Of these drug names, a consensus or majority structure was determined for 91% of compounds.

**Figure 4:**
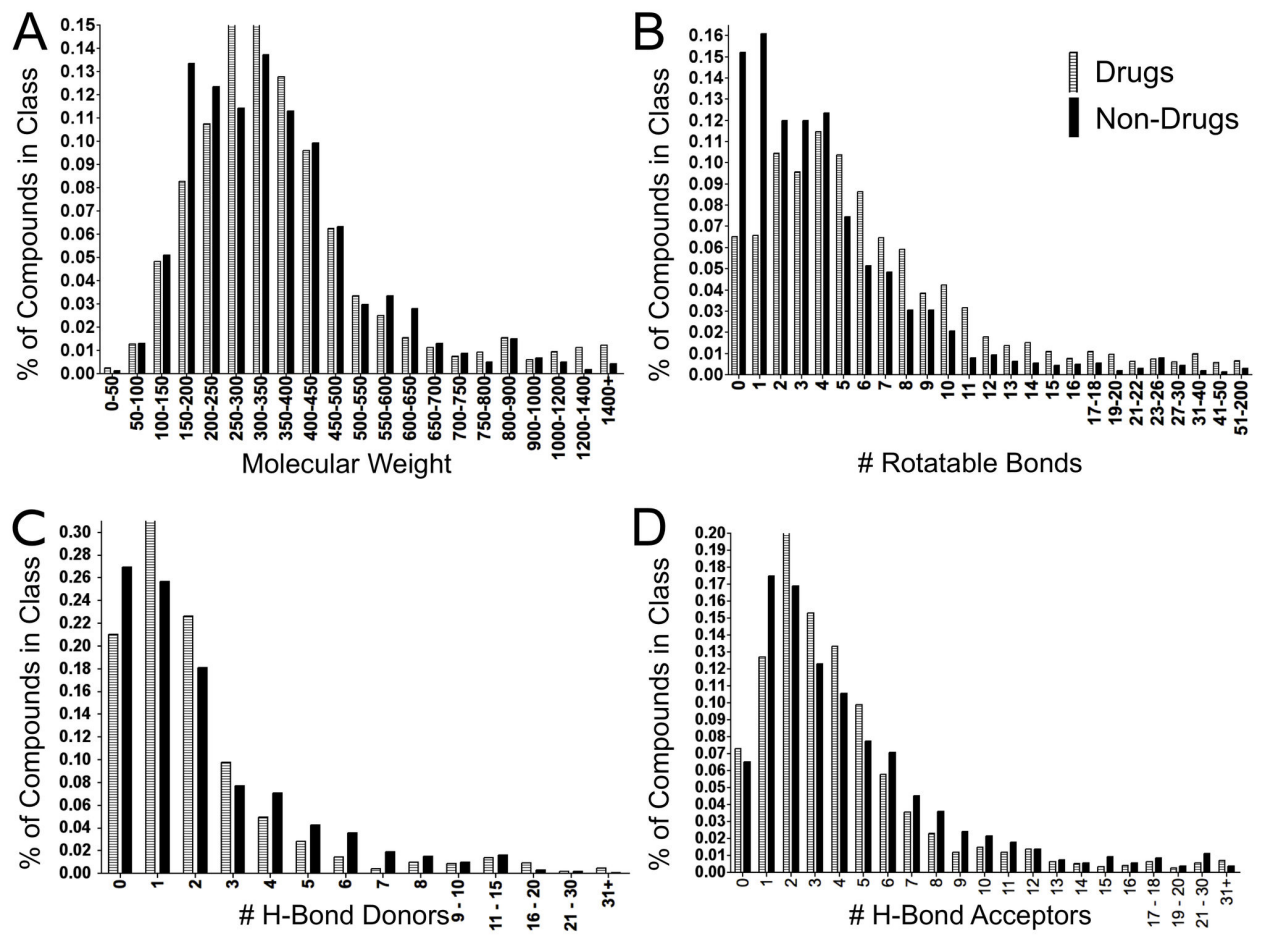
‘Drug-like’ properties of approved drugs vs. non-approved compounds in SWEETLEAD. Comparison of molecular descriptors frequently referenced as important to drug-likeness between approved drugs and other compounds in the SWEETLEAD database. The property distributions for both the approved drugs and non-approved compounds in SWEETLEAD are shown for A) molecular weight, B) the number of rotatable bonds, C) the number of hydrogen bond donors and D) acceptors.

 The SWEETLEAD database was then constructed by inputting this list of drug and chemical names into our structure determination algorithm. For most compound names, either a consensus or majority structure was determined. Figure 3 shows the outcomes of the 1996 API names obtained from the FDA electronic Orange Book. Over 90% of names led to the identification agreed upon structure, and consensus between all databases polled was achieved for 55% of compound names. From the combined list of drug and chemical names, 4442 chemical structures were collected, and the classification of all compounds included in the initial release of SWEETLEAD is given in Table 1. Of the 4442 compounds, 2836 are approved drugs in at least one region and 1427 are FDA approved drugs. 217 of these compounds are scheduled drugs in the United States, and 1625 of the compounds derive from traditional medicinal herbs. Some compounds can be multiply classified, such as methamphetamine and codeine which are both approved therapeutics and scheduled drugs. Reflecting the fact that multiple formulations of APIs are combined into a single entry in SWEETLEAD, the number of unique PubChem IDs (4713) and ChemSpider IDs (4501) is greater than the total number of unique compounds. The entire SWEETLEAD database can be downloaded at https://simtk.org/home/sweetlead. A natural question following our decision to include approved and non-approved compounds in SWEETLEAD, and in light of often discussed ‘drug-like’ properties, would be whether the two sets of compounds are similar in their molecular properties. Figure 4 compares the distributions of molecular properties of the approved drugs and non-approved chemicals in the SWEETLEAD database. Molecular weight and rotor, hydrogen bond donor, and hydrogen bond acceptor distributions are shown, while predicted LogP was omitted due to inaccuracies in computational LogP prediction tools. Chi-Squared tests indicate that the distributions for all for properties are statistically different between the drug and non-drug groups, however this difference is most marked for the distributions of rotatable bonds where over 31% of non-drugs have less than 2 rotatable bonds as compared to 13% for approved drugs. Additionally, 93% of the non-approved compounds pass Lipinski’s Rule of 5 with only 1 violation, identical to the 93% mark for approved drugs, which indicates their drug likeness according to that metric. Overall, both the drugs and non-drugs in the SWEETLEAD database exhibit molecular properties similar to the ‘drug-like’ rules of thumb typically used in a drug discovery setting.

**Table 1:** SWEETLEAD Database Content Profile.

SWEETLEAD Data Field	Number Unique Entries
Total Molecules	4442
Approved Drugs	2836
Illegal/Scheduled Drugs	217
Herbal Isolates	1625
PubChem IDs	4713
KEGG IDs	2592
ChemSpides IDs	4501
DrugBank IDs	1286
ChEBI IDs	1760
PharmGKB IDs	1070

## Conclusions

In summary, SWEETLEAD is a highly curated database containing accurate chemical structures and identification information for the world’s known approved drugs and other non-toxic chemicals. We hope to continue to refine and build the SWEETLEAD database by adding to the number of compounds represented by the database, increasing the quantity of external database links provided herein, and correcting any errors which exist in the present release. Given the sensitivity of computational based approaches to data quality, it is our hope that SWEETLEAD will serve as a valuable resource supporting cheminformatics based repurposing efforts. 

## Supporting Information

Figure S1 **Example Consensus Building for Apomorphine Hydrochloride.** Step 1) To begin the consensus building process, each Reference Database is queried by the compound name, and Structural Database IDs are collected. The resulting IDs are tallied by assigning each Structural Database ID a vote equal to 1/(# Structural Database IDs returned by that Reference Database). So, in this example, PubChem ID 107882 is the most popular PubChem ID by obtaining 1.25 votes (1 for being the only result returned by PubChem, and .25 for being one of 4 IDs returned by ChemSpider). Step 2) The structure associated with each winning Structural Database ID is obtained, and standardized according to the text. The structures are then associated with all Structural Database IDs which return the structure after standardization (various salt forms, protonation states, etc.) Step 3) The most popular structure is identified. In this case, there is a consensus structure as all Reference Databases pointed towards Structural Database IDs matching the most popular structure. Step 4) The winning structure is associated with the drug name, winning Structural Database IDs, synonyms associated with the winning Structural Database IDs, and entered into the SWEETLEAD database.
(PDF)
