# Supplementary material for: SWEETLEAD: an In Silico Database of Approved Drugs, Regulated Chemicals, and Herbal Isolates for Computer-Aided Drug Discovery
Source: PLoS One. 2013 Nov 1;8(11):e79568. doi: 10.1371/journal.pone.0079568 (PMC3815155; doi:10.1371/journal.pone.0079568)
Supplement: Figure S1 — Example Consensus Building for Apomorphine Hydrochloride. Step 1) To begin the consensus building process, each Reference Database is queried by the compound name, and Structural Database IDs are collected. The resulting IDs are tallied by assigning each Structural Database ID a vote equal to 1/(# Structural Database IDs returned by that Reference Database). So, in this example, PubChem ID 107882 is the most popular PubChem ID by obtaining 1.25 votes (1 for being the only result returned by PubChem, and .25 for being one of 4 IDs returned by ChemSpider). Step 2) The structure associated with each winning Structural Database ID is obtained, and standardized according to the text. The structures are then associated with all Structural Database IDs which return the structure after standardization (various salt forms, protonation states, etc.) Step 3) The most popular structure is identified. In this case, there is a consensus structure as all Reference Databases pointed towards Structural Database IDs matching the most popular structure. Step 4) The winning structure is associated with the drug name, winning Structural Database IDs, synonyms associated with the winning Structural Database IDs, and entered into the SWEETLEAD database. (PDF) [file pone.0079568.s001.pdf]

Step 1: Get Database IDs, and rank

Database Queried	PubChem ID(s)	KEGG ID(s)	ChemSpider ID(s)	DrugBank ID(s)	CHEBI ID(s)
**PubChem**	71313467				
**KEGG**	107882	D02004		DB00714	
**ChemSpider**	5702010, 10782, 9410, 6852389		4642402, 97017, 9039, 5254711, 23099928		31228
**DrugBank**	6005	D07460		DB00714	48538
**PharmGKB**					
**Wikipedia**					
**Winner(s)**	**107882 (1.25 votes)**	**D02004, D07460 (Tie)**	**5 way tie**	**DB00714**	**31228, 48538 (Tie)**

Step 2: Associate winning database IDs with standardized structures

Structure	PubChem ID(s)	KEGG ID(s)	ChemSpider ID(s)	DrugBank ID(s)	CHEBI ID(s)
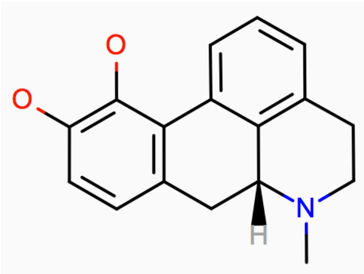	107882	D07460, D02004	9039, 2309928, 97017	DB00714	31228, 48538
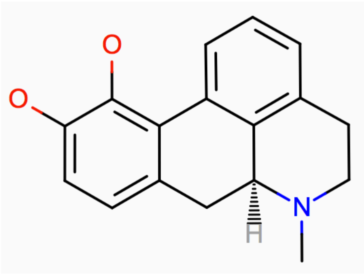			5254711		
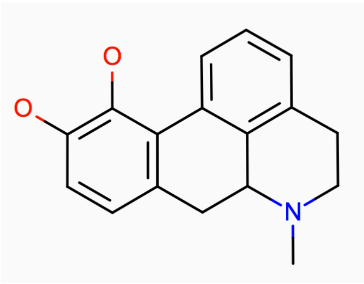			4642602		

Step 3: Identify structure suggested by each database's winners (consensus)

Structure	PubChem ID(s)	KEGG ID(s)	ChemSpider ID(s)	DrugBank ID(s)	CHEBI ID(s)
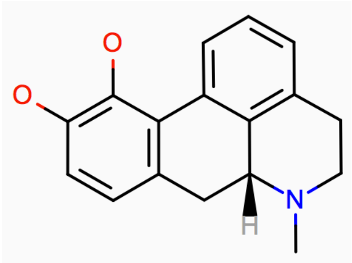	107882	D07460, D02004	9039, 2309928, 97017	DB00714	31228, 48538

Step 4: Combine Apomorphine Hydrochloride results with Apomorphine results, since both return the same structure

Structure	PubChem ID(s)	KEGG ID(s)	ChemSpide r ID(s)	DrugBank ID(s)	CHEBI ID(s)
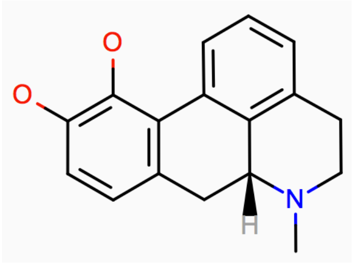	6005, 107882	D07460, D02004	5783, 9039, 2309928, 97017	DB00714	31228, 48538
